# Atmospheric Carbon Dioxide Modifies the Antimicrobial Activity and Oxidative Stress Generated by Ciprofloxacin in *Escherichia coli*

**DOI:** 10.3390/pathogens14070689

**Published:** 2025-07-14

**Authors:** Viviana Cano Aristizábal, Elia Soledad Mendoza Ocampo, Melisa de los Ángeles Quinteros, María Gabriela Paraje, Paulina Laura Páez

**Affiliations:** 1 Department of Pharmaceutical Sciences, Faculty of Chemical Sciences, National University of Córdoba, Córdoba X5000HUA, Argentina; vcanoa87@gmail.com (V.C.A.); elia.mendoza@unc.edu.ar (E.S.M.O.); mquinteros@unc.edu.ar (M.d.l.Á.Q.); 2 Pharmaceutical Technology Research and Development Unit (UNITEFA), National Scientific and Technical Research Council (CONICET), Córdoba X5000HUA, Argentina; 3 Faculty of Exact, Physical, and Natural Sciences, National University of Córdoba, Córdoba X5000HUA, Argentina; gabrielaparaje@gmail.com; 4 Multidisciplinary Institute of Plant Biology (IMBIV), National Scientific and Technical Research Council (CONICET), Córdoba X5000HUA, Argentina

**Keywords:** carbon dioxide, ciprofloxacin, *Escherichia coli*, reactive oxygen species, reactive nitrogen species, antioxidant mechanisms

## Abstract

The accelerated increase in atmospheric CO_2_ concentration is one of the most pressing problems at present. It is possible that this increase causes slight modifications in intracellular CO_2_. The aim of this work was to determine whether CO_2_ at different concentrations can affect the oxidative damage caused by ciprofloxacin (CIP) in *Escherichia coli* and to evaluate the possible implications of this effect for human health. To identify the effects of CO_2_ on the action of CIP, reactive oxygen (ROS) and reactive nitrogen (RNS) species were measured at two different CO_2_ concentrations while monitoring the bacterial antioxidant response. These assays showed that CO_2_ led to a decrease in ROS formation relative to that under atmospheric conditions (ACs), while it had the opposite effect on RNS formation, which increased relative to that under ACs. Under CO_2_ conditions, antioxidant defenses were less activated, with superoxide dismutase, catalase, and ferric reducing assay potency decreasing compared to those under ACs; however, reduced glutathione exhibited the opposite behavior. In the presence of CO_2_, the activity of CIP against *E. coli* was reduced relative to that under ACs. In conclusion, CO_2_ interferes with the action of CIP in bacterial cells, generating changes in oxidative stress.

## 1. Introduction

Atmospheric carbon dioxide (CO_2_) levels have become an important issue for the scientific community due to their significant contribution to global warming. The levels of CO_2_ in the atmosphere have increased significantly in recent decades due to human activities such as the burning of fossil fuels, deforestation, and industrialization. The rate of increase in atmospheric CO_2_ over the past 70 years is almost 100 times greater than at the end of the last Ice Age. The global average annual concentration of CO_2_ in the atmosphere has increased by more than 40% since the beginning of the Industrial Revolution, from 280 ppm in the mid-18th century to 402 ppm in 2016. The average ambient concentration of CO_2_ (in fresh air) has rapidly increased and currently fluctuates around 410 ppm [1]. This increase in atmospheric CO_2_ concentration has implications not only for atmospheric and climatic processes, but also for biological systems. CO_2_ is a major by-product of cellular metabolism and constitutes the main physiological pH buffer system in eukaryotes. It is also necessary for the growth of many microorganisms [2,3].

The effects of CO_2_ on cell metabolism have received little research attention. Moreover, it has been shown that both endogenous and exogenous CO_2_ alter the growth kinetics of enteropathogenic *Escherichia coli* (EPEC), and bicarbonate enhances the in vitro activity of kanamycin and gentamicin against EPEC [4]. While atmospheric CO_2_ concentrations are increasing rapidly, they are still low compared to plasma CO_2_ concentrations (50,000 ppm or 5%) [5]. However, previous studies have shown that an increase in the atmospheric concentration of CO_2_ (1–10%) affects biochemical reactions at the cellular level, leading to an increase in intracellular oxidative stress in human neutrophils [6], lung inflammation in mice [7], and the increased virulence of different bacterial pathogens [8,9].

Oxidative stress is caused by exposure to reactive oxygen species (ROS) such as superoxide anions (O_2_^•−^), hydrogen peroxide (H_2_O_2_), and hydroxyl radicals (HO^•^). ROS can be harmful to biomolecules and cause oxidative damage, which is implicated in various pathologies (neurodegenerative diseases, atherosclerosis, cancer, and other disorders). However, they play a crucial role in homeostasis, cellular signaling, the regulation of metabolism, and memory formation through DNA methylation [10]. A mechanism was proposed in which the alteration of the bacterial membrane triggers envelope stress, which subsequently disrupts the anaerobic response regulatory system, accelerating cellular respiration [11]. The hyperactivation of the electron transport chain induces the formation of superoxide and hydrogen peroxide, damaging iron–sulfur groups, thereby releasing ferrous iron. The released iron can then react with hydrogen peroxide via the Fenton reaction, generating hydroxyl radicals that can directly damage DNA, lipids, and proteins or oxidize the pool of deoxynucleotides, indirectly damaging DNA. However, this theory has recently become the subject of much debate [12,13].

Up to 1–2% of the oxygen consumed by a cell can be converted into oxygen radicals, which can lead to ROS production. The main source of ROS in vivo is aerobic respiration. However, ROS are also produced by the peroxisomal β-oxidation of fatty acids, microsomal cytochrome P450, xenobiotic compound metabolism, the stimulation of phagocytosis by pathogens or lipopolysaccharides, arginine metabolism, and tissue-specific cellular enzymes [14,15]. To counteract oxidative stress, cells must first identify the ROS produced and transduce signals to increase their enzymatic and non-enzymatic antioxidant defenses, such as superoxide dismutase (SOD), catalase (CAT), and reduced glutathione (GSH) [16,17]. The generation of intracellular ROS and their local redox state are important for understanding cellular pathophysiology. Some subcellular compartments are more oxidizing (such as the endoplasmic reticulum (ER), lysosomes, and peroxisomes), while others are more reducing (mitochondria and nuclei). Therefore, ROS levels can fluctuate between subcellular compartments and can lead to beneficial or pathological effects [18]. The superoxide anion and its derivatives—hydrogen peroxide and the hydroxyl radical—are the main active oxygen-containing chemical species. Although ROS are essential for some cellular processes, such as transcription factor activation, gene expression, and protein phosphorylation, their uncontrolled production leads to indiscriminate oxidative attack, affecting the inflammatory response, proteins, and lipids and ultimately causing cell death and organ damage [19,20].

In organisms, ROS are regularly generated, both endogenously by the respiratory chain in aerobic metabolism and exogenously by different external factors, such as exposure to radiation, light, metals, and antibiotics [21], affecting bacterial genera with different types of oxidative metabolism [22]. This may be of key importance, as bacteria leave the relatively low CO_2_ levels of the external atmosphere for the higher CO_2_ levels found in most multicellular host organisms. Bacteria may upregulate virulence factors at the host’s physiological CO_2_ levels (as opposed to atmospheric CO_2_ levels) in order to facilitate colonization or infection [23].

In recent years, some antibiotics have been characterized as stimulators of oxidative stress, including ciprofloxacin (CIP), which is known to interfere with the replication and transcription of deoxyribonucleic acid (DNA) by inhibiting DNA gyrase/topoisomerase II and topoisomerase IV [24]. However, this is not the only mechanism of action since CIP has been proven to induce ROS formation, causing increased O_2_^•−^ levels in *Staphylococcus aureus*, *E. coli*, and *Pseudomonas aeruginosa*. It has been found that CIP increases ROS in susceptible strains of *S. aureus*, producing a state of oxidative stress, but not in resistant strains [22,25,26].

The aim of this work was to determine whether high CO_2_ atmospheric concentrations can affect the oxidative damage generated by CIP in *E. coli* and to evaluate their possible implications for human health.

## 2. Materials and Methods

Chemicals and reagents. Luria–Bertani (LB) media (MP, USA), Nitro blue tetrazolium (NBT), 2′,7′-dichlorodihydrofluorescein diacetate (H_2_-DCFDA), and N-(1-naphthyl) ethylenediamine dihydrochloride were all obtained from Sigma-Aldrich (St. Louis, MO, USA). Sulfanilamide was obtained from Merck (Darmstadt, Germany). CIP was obtained from Todo Droga (Córdoba, Argentina).

Experimental CO_2_ conditions. Carbon dioxide was purchased commercially from the AIR PRODUCTS gas company (Indura group, Buenos Aires, Argentina) with the following concentrations: 50 ppm CO_2_ (equivalent to 0.005%), 20% O_2_, balance (BLCE) N_2_ 8 m^3^; and 50.000 ppm CO_2_ (equivalent to 5%), 20% O_2_, BLCE N_2_ 8 m^3^. To carry out the experiments in the presence of CO_2_, 1 mL of the overnight suspension was added to 24 mL of LB in each erlenmeyer for the assays (Figure S1, Supplementary Materials).

Survival curves in controlled concentrations of CO_2_. This assay was performed with a total incubation time of 8 h. *E. coli* ATCC 25922 was cultivated aerobically in LB broth with stirring at 140 rpm for 18 h at 37 °C. Then, the bacterial cells were exposed to different CO_2_ concentrations (50 and 50,000 ppm) and ACs in the presence of different concentrations of CIP (0, 0.5, and 50 µg/mL). Serial dilutions 1:10 of bacterial suspensions were prepared in phosphate buffer (PBS) 0.05 M, pH 7.2, and plated on LB agar. After 18 h of incubation at 37 °C, colony-forming units (CFU) were counted and the results are expressed as CFU/mL [27,28].

Determination of ROS. The kinetics of ROS generation in *E. coli* ATCC 25922 treated with CIP were quantified by spectrofluorometry using H_2_-DCFDA as a fluorescent probe (480 nm and 520 nm were used as excitation and emission wavelengths, respectively) [29]. The bacterial cells were exposed to different CO_2_ concentrations (50 and 50,000 ppm) and ACs in the presence of different concentrations of CIP (0, 0.5, and 50 µg/mL) for 3 h of incubation. Then, 20 μL of a 20 µM H_2_-DCFDA aqueous solution was added. The fluorescence intensity was measured 30 min later with a spectrofluorometer (Biotek Synergy HT, Santa Clara, CA, USA). These results were expressed as arbitrary fluorescence units (a.u) per CFU/mL [30]. Non-treated bacterial suspensions were used as the control. The experiments were carried out in triplicate.

Quantification of reactive nitrogen species (RNS). The generation of nitric oxide in *E. coli* ATCC 25922 was quantified by the Griess reaction according to the methodology described by Guevara et al., where N-(1-Naphthyl)ethylenediamine and sulfanilamide are used to form a diazonium salt, which is then measured spectrophotometrically [31,32]. First, the bacterial suspension was incubated for 3 h with CIP under CO_2_ conditions, as previously described. Then, 100 µL of each sample was mixed with 50 μL of 2% sulfanilamide in 5% (*v*/*v*) HCl and 50 μL of 0.1% N-(1-naphthyl)ethylenediamine dihydrochloride aqueous solution. Fifteen minutes later, the formation of the azo dye was measured spectrophotometrically at 543 nm. The absorbance was directly proportional to the nitrite content of the standard solution. These results are expressed as µM of sodium nitrate per mg of protein (µM NaNO_2_/mg of protein).

Ferric reducing assay potency (FRAP). In total, 50 µL of the bacterial suspension was incubated with 150 µL of a mixture of 3.1 mg/mL 2,4,6-tripyridyl-1,3,5-triazine (TPTZ) in 40 mM HCl, 5.4 mg/mL FeCl_3_·6H_2_O, and 300 mM acetate buffer (pH 3.6). Absorbance was read at 593 nm at three different times, with samples taken at 0, 2, and 4 h. The results are expressed as µM of Fe^2+^ per mg protein [33].

Superoxide dismutase activity. An overnight culture of *E. coli* was prepared in LB media. Afterward, 1 mL of the bacterial suspension (OD_600_ = 1) was incubated with CIP (0.5 and 50 µg/mL) and without CIP under ACs and different concentrations of CO_2_ (50 and 50,000 ppm) in 24 mL of LB broth at 37 °C. Samples were taken at 0, 2, and 4 h of incubation, centrifuged at 13,000 rpm for 15 min, and the supernatant (extracellular SOD) was separated. The pellet was resuspended in 0.5 mL of PBS (intracellular SOD). The reaction mixture was obtained by incubating 100 µL of the intracellular or extracellular fraction, 100 µL of 75 µM NBT in DMSO, 300 µL of 13 mM methionine, 300 µL of 100 nM EDTA, and 300 µL of 2 M riboflavin in 50 mM PBS pH 7.8. Then, the samples were exposed to 20 W fluorescent lights for 6 min to trigger the reaction. The final color was measured spectrophotometrically at 560 nm. These assays were performed for 4 h, and the results were expressed as SOD units per mg of protein (USOD/mg of protein) [34,35].

Catalase determination. CAT activity in *E. coli* was determined using a spectrophotometric method with potassium dichromate in acidic solution. An overnight culture of *E. coli* was prepared in LB media. Then, 1 mL of the bacterial suspension (OD_600_ = 1) was incubated with CIP (0.5 and 50 µg/mL) and without CIP under AC and controlled concentrations of CO_2_ (50 and 50,000 ppm) in 24 mL of LB broth at 37 °C. The reaction was followed for 4 h. Then, 2 mL of 0.2 M H_2_O_2_ solution and 2.5 mL of PBS pH 7 were added to 1 mL of each sample. After that, 1 mL was removed and mixed with 2 mL of reagent (2% potassium dichromate in glacial acetic acid). These samples were incubated at 100 °C for 2 min and then cooled in an ice bath. Then, the absorbance was determined at 570 nm. The results are expressed as UCAT per mg protein. A UCAT is the amount of enzyme that reacts with 1 μM H_2_O_2_ per min at 25 °C to pH 7 [33,34].

Assay of GSH. The Ellman reagent (5,5-dithiobis-2-nitrobenzoic acid) was used to form a colored compound in the presence of GSH, which is read spectrophotometrically at 412 nm. An overnight culture of *E. coli* ATCC 25922 in LB broth was prepared. Then, 1 mL of the bacterial suspension (OD_600_ = 1) was incubated with CIP (0.5 and 50 µg/mL) and without CIP for 4 h under AC and controlled concentrations of CO_2_ (50 and 50,000 ppm) in 24 mL of LB broth. These samples were taken at 0, 2, and 4 h. Then, 100 μL of each sample was incubated at room temperature with 20 µL of glutathione reductase (6 U/mL), 50 µL of NADPH (4 mg/mL), and 20 µL of 5,5-dithiobis-2-nitrobenzoic acid (DTNB) (1.5 mg/mL). The results are expressed as mM of GSH per mg protein [35,36].

Statistical analysis. Data are expressed as the mean ± standard deviation (SD) of three independent experiments carried out under identical conditions. They were subjected to one-way analysis of variance (ANOVA) and a subsequent Bonferroni test using the Graph Pad Prism 8 statistical software. The confidence limit used was 0.05.

## 3. Results

### 3.1. Survival Curves in Controlled Concentrations of CO_2_

**Figure 1:**
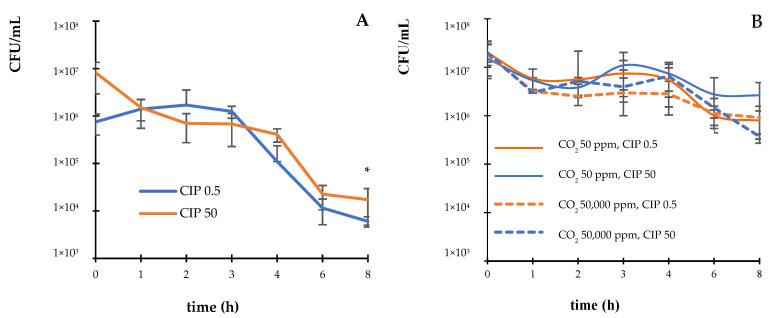
Survival curves (CFU/mL) of *E. coli* ATCC 25922 incubated with CIP (**A**) under atmospheric conditions and (**B**) controlled concentrations of CO_2_ (50 ppm and 50,000 ppm). The error bar indicates the SD. * *p* < 0.05 compared to the initial time of the assay.

The kinetics of the bactericidal activity of CIP at different CO_2_ concentrations (50 and 50,000 ppm) against *E. coli* ATCC 25922 were examined. The results revealed that CIP at the MIC (0.5 µg/mL) and supra-MIC (50 µg/mL) exerted bactericidal activity against the *E. coli* strain, decreasing the CFU/mL by three orders of magnitude within 6 h of exposure (Figure 1A). In contrast, in the presence of CO_2_, the bactericidal effect of CIP was not observed until after 8 h of incubation (Figure 1B).

### 3.2. Determination of ROS and RNS

**Figure 2:**
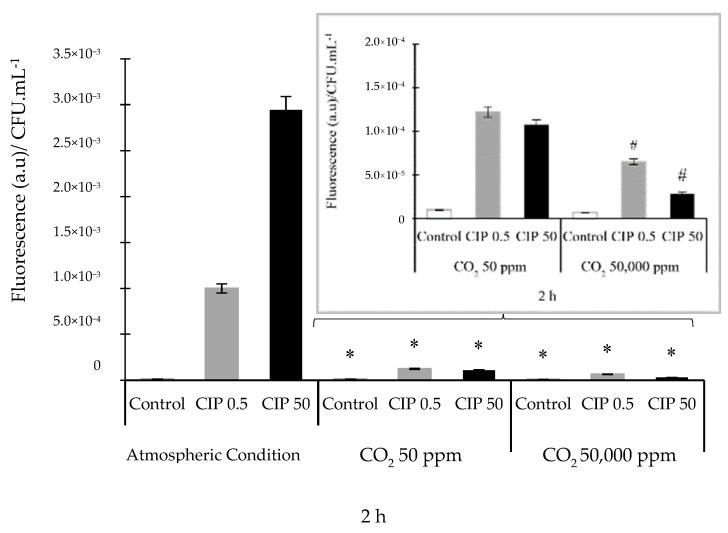
ROS determination using the spectrofluorometric assay with H_2_-DCFDA in *E. coli* ATCC 25922 incubated under different atmospheric conditions with (0.5 and 50 µg/mL) and without (control) CIP. The inset shows the results of CIP action in the presence of CO_2_ (50 ppm and 50,000 ppm). The error bar indicates the SD. * *p* < 0.05 relative to atmospheric conditions. # *p* < 0.05 between CIP and CO_2_ concentrations.

ROS were produced within the first 2 h after the CIP treatment, both in ACs and controlled CO_2_ concentrations. It was observed that, under ACs and in the presence of CIP, O_2_^•^¯ was the most abundant species at this time point. Simultaneously, a low amount of NO was observed. When experiments were performed in the presence of CO_2_ and CIP, the activity of HO^•^ and NO compounds increased (see Supplementary Materials, Figures S2–S6). For this reason, the results were analyzed at that time. The two CIP concentrations studied under ACs enhanced the formation of ROS, while CIP under CO_2_ conditions significantly decreased the formation of ROS relative to that under ACs (*p* ˂ 0.05). These ROS decreases were (6.06 ± 0.10) × 10^−5^ and (3.00 ± 0.15) × 10^−5^ (a.u)/CFU·mL^−1^, which are equivalent to 93 and 99% at 0.5 and 50 μg/mL of CIP, respectively, compared to levels under ACs (Figure 2). The significant (*p* < 0.05) decrease in ROS levels was dependent on the concentrations of CIP and CO_2_ (Figure 2, inset).

As shown in Table 1, in ACs, CIP does not induce an increase in RNS formation relative to the control. However, at 50 ppm of CO_2_ and in the presence of CIP, the levels of RNS formed were greater than those under ACs. A significant increase compared to the control (*p* < 0.05) and ACs (*p* < 0.05) was observed; these increases were approximately 84.985 ± 0.011 and 81.921 ± 0.003 µM NaNO_2_/mg of protein for 0.5 and 50 µg/mL of CIP, respectively, compared to levels under ACs. In contrast, at 50,000 ppm of CO_2_, only RNS formation increased; the synergic effect observed between CIP and 50 ppm of CO_2_ was not observed at this concentration.

### 3.3. Enzymatic and Non-Enzymatic Antioxidant Activity

**Figure 3:**
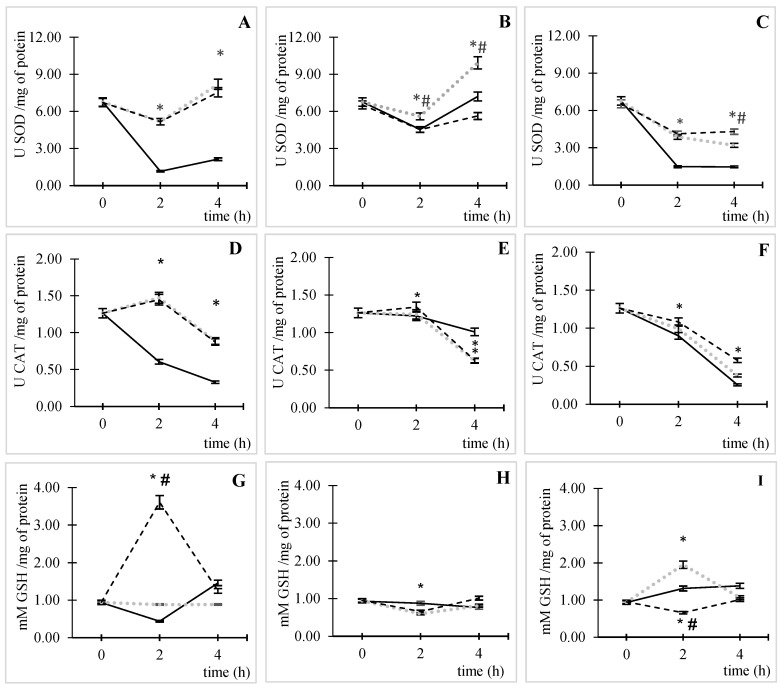
Enzymatic and non-enzymatic antioxidant activity determination in *E. coli* ATCC 25922. Activation kinetics of SOD in different conditions [(**A**) ACs, (**B**) 50 ppm of CO_2_, and (**C**) 50,000 ppm of CO_2_], activation kinetics of CAT in different conditions [(**D**) ACs, (**E**) 50 ppm of CO_2_, and (**F**) 50,000 ppm of CO_2_], and activation kinetics of CAT in different conditions [(**G**) ACs, (**H**) 50 ppm of CO_2_, and (**I**) 50,000 ppm of CO_2_], incubated without CIP (black line), with 0.5 µg/mL of CIP (dotted line in gray color), and with 50 µg/mL of CIP (hyphen line). The error bar indicates the SD. * *p* < 0.05 relative to the control. # *p* < 0.05 between CIP concentrations.

Under ACs and without CIP, an increase in enzymatic (Figure 3A,B) and non-enzymatic antioxidant activity (Figure 3G) was observed. Figure 3A shows the SOD results obtained under ACs; in the absence of CIP (control), enzyme consumption increased until 2 h of incubation, after which it remained relatively constant over time. In the presence of CIP, this effect was inverted; enzyme activation increased after 2 h of incubation at both CIP concentrations (5.17 ± 0.06 U SOD/mg of protein), equivalent to a four-fold increase in SOD activity compared to the basal condition (*p* ˂ 0.05). At 4 h of incubation, SOD activity differed from that under basal conditions at both CIP concentrations (*p* ˂ 0.05).

A similar effect was observed at high CO_2_ concentrations (Figure 3C). Nevertheless, the SOD enzyme activity decreased over time in the presence of CIP (*p* < 0.05). At low CO_2_ concentrations (Figure 3B), there was a reduction in enzymatic activity until an incubation time of 2 h for the three studied variables. This reduction was very similar to the control conditions without and with 50 µg/mL of CIP; then, the activity of the enzyme recovered at 4 h of incubation, and it was significantly different from that under control conditions only at 0.5 µg/mL of CIP (*p* ˂ 0.05).

Under ACs (Figure 3D), at 2 h of incubation, CAT enzyme activity was 1.5 ± 0.04 and 1.4 ± 0.02 U CAT/mg of protein for 0.5 and 50 µg/mL of CIP relative to the control, which is equivalent to increases of 143 and 139%, respectively (*p* ˂ 0.05). This time coincided with the highest levels of ROS formation.

At controlled CO_2_ concentrations, the enzyme activity increased only at 50 ppm and 50 µg/mL of CO_2_ and CIP, respectively, at the time of maximum ROS production (Figure 3E), with an activity of 1.3 ± 0.02 UCAT/mg of protein, which corresponds to a 10% increase relative to the control (*p* ˂ 0.05). In contrast, at 50,000 ppm of CO_2_, a reduction in enzymatic activity was observed throughout the duration of the assay (Figure 3F). It should be noted that at 4 h of incubation, in all cases, a gradual reduction in enzymatic activity was observed, which was more pronounced at high CO_2_ concentrations (Figure 3F; *p* < 0.05).

The GSH antioxidant capacity differed among all tested conditions. In ACs (Figure 3G) at 50 µg/mL of CIP, a significant increase in GSH was observed relative to the control (*p* < 0.05) at the time of maximum ROS formation (2 h), while at the lowest CIP concentration, there was no significant change at any time point in the assay. At 50 ppm of CO_2_ (Figure 3H), at 2 h of incubation, GSH was consumed (0.13 ± 0.03 mM GSH/mg of protein) in *E. coli* due to the action of 50 µg/mL CIP relative to the control without CIP (*p* < 0.05).

At high concentrations of CO_2_ (Figure 3I), different behaviors were observed depending on the CIP concentration. At 0.5 µg/mL of CIP, relative to the control without CIP, an increase in GSH (1.95 ± 0.04 mM GSH/mg of protein) was observed at 2 h of incubation (*p* < 0.05), whereas at 50 µg/mL of CIP, enzyme consumption (0.66 ± 0.02 mM GSH/mg of protein) was observed at 2 h relative to the control (*p* < 0.05). In addition, at 4 h of incubation, there was a gradual reduction in GSH activity under ACs and 50 ppm of CO_2_. However, at 50,000 ppm of CO_2_, the high CIP concentration enhanced GSH activity, while the low CIP concentration (0.5 µg/mL) produced the opposite result.

In CO_2_ conditions, SOD and CAT activities were much lower than those in ACs. This can be attributed to the decline in ROS formation due to the interaction between CIP and CO_2_, which led to less activation of antioxidant defenses to neutralize these species. Nevertheless, GSH was the species that had the most significant increase in the presence of CO_2_; this behavior depended on the CIP and CO_2_ concentrations.

At 50 ppm of CO_2_, the increase in GSH formation was 121% at 0.5 µg/mL of CIP relative to ACs, while 50 µg/mL of CIP led to the opposite result (82% consumption). At 50,000 ppm of CO_2_, both CIP concentrations led to GSH consumption relative to that under ACs (32 and 96% for 0.5 and 50 µg/mL of CIP, respectively). This behavior could be related to the decrease in ROS formation observed in the presence of CO_2_.

FRAP in *E. coli* was studied over a period of 2 h (maximum stimulus of ROS); the results are shown in Table 2, where it can be seen that, in ACs, there was a marked decrease in FRAP in the presence of CIP (Table 2, first column). However, this effect was modified by CO_2_, whose presence alone induced a large decrease in FRAP. At 50 ppm of CO_2_, the FRAP value in the presence of CIP was similar to that in ACs (Table 2, first column), while, at 50,000 ppm of CO_2_, the decrease was not as pronounced, but adding CIP had a substantial effect; the presence of this ATB contributes to stimulating the antioxidant capacity, reaching the same values as those observed under ACs.

## 4. Discussion

The bactericidal activity of CIP against *E. coli* was not evident in the presence of CO_2_ until after 8 h of incubation. These results are opposite to the results reported by Farha et al., who studied the effect of bicarbonate (HCO_3_^−^) as an enhancer of CIP activity against *E. coli* and concluded that the HCO_3_^−^ buffer system is effective in promoting CIP’s antimicrobial activity [37], indicating that the equilibrium of the buffer system was perturbed in the opposite manner. Previous reports have shown that pH variation can increase or decrease (at high and low pH, respectively) CIP’s bactericidal activity [38,39,40]. This could be related to the protonated/unprotonated CIP structure [41,42] since it is known that the chemical environment generated by ionic carboxylic acid and carbonyl groups in the 3 and 4 positions in CIP, respectively, is necessary to form strong hydrogen bonds with DNA and/or to coordinate with Mg (II) cations, making these groups essential to antibacterial activity [43].

It is known that CIP leads to the accumulation of oxygen species inside bacterial cells as a secondary mechanism of action [44].

Nevertheless, we studied the HO^•^, O_2_^•^¯, and NO formation pathways using the scavengers 2,2′-bipyridyl, Tyron, and carboxy-PTIO, respectively, which make it possible to identify radical species that may be affected by CO_2_ through a decrease in ROS or RNS formation (depending on the species being studied); these effects can be determined by measuring products of the reaction between the reactive species and the scavengers and comparing the results with those under ACs [45].

The results obtained in ACs are in agreement with previously published articles, which showed that ROS formation was quickly induced by CIP in *Proteus mirabilis* and *S. aureus.* These results confirm that ROS are involved not only in the toxicity but also in the mechanism of action of CIP [33,46]. Masadeh et al. observed the same behavior in a different reference strain [47]; however, none of these studies included an evaluation in a CO_2_-modified atmosphere. 

The decrease in ROS formation generated by CIP in the presence of CO_2_ could potentially be explained by RNS formation. These alterations in RNS formation in the presence of CO_2_ and CIP could favor the cytoprotective behavior of NO described by Wink and Mitchell, who indicated that NO can neutralize ROS and, in turn, critically alter biomolecules such as enzymes and DNA, depending on both the NO concentration and the organism under study [48,49]. Additionally, Salgo et al. determined that NO concentrations between 0.05 and 8 mM favor ONOO^−^ formation, which could cause the aforementioned damage to biomolecules. Adding to these findings, Salgo et al. determined that NO concentrations between 0.05 and 8 mM favor ONOO^•−^ formation, which could cause DNA lesions and induce cell death [50]. Nevertheless, the highest NO concentrations obtained in this work were 85 µM, which is not high enough to cause DNA damage and cell death. The interaction between ROS and RNS is complex. ROS can react with NO to form RNS, such as peroxynitrite, which can have different biological implications compared to ROS alone. The elevated levels of RNS in the presence of CO_2_ suggest a potential cross-talk where CO_2_ concentration modifies the balance between oxidative and nitrosative stress. We observed that the effect of CIP and low CO_2_ was not replicated at high CO_2_, which suggests a threshold effect where higher CO_2_ might suppress certain signaling pathways or metabolic processes. This could dampen the production of both ROS and RNS, suggesting that CO_2_ levels critically influence the oxidative and nitrosative stress in bacteria. Our findings suggest that CO_2_ modulates the balance between ROS and RNS in *E. coli* under CIP treatment, with distinct behaviors observed at different CO_2_ concentrations. Understanding these interactions will be crucial for fully appreciating the adaptive responses of bacteria to oxidative and nitrosative stress, potentially guiding therapeutic strategies against antibiotic resistance.

These results indicate that CO_2_ affects the activation of antioxidant defense systems since, in ACs, all antioxidant defenses were activated at the time point where maximum ROS formation was observed. This behavior is similar to that reported by other authors who evaluated CIP’s capacity to induce ROS formation through these defenses (GSH, ascorbic acid, SOD, and CAT) in *E. coli* [44]. This behavior has also been described for other bacterial genera, such as *P. aeruginosa*, *Proteus mirabilis*, and *S. aureus* [26,27,45]. Goswami et al. showed that GSH reduced CIP’s antibacterial effect by counteracting the associated oxidative stress and promoting its exit from the cell [46]. This behavior has been reported by other authors, not only in *E. coli* [46] but also in other bacterial species [26,27,45].

CIP’s capacity to form ROS in *E. coli* was reduced by CO_2_; therefore, reactive species at low concentrations can be neutralized by RNS or GSH, which may explain the observed decline in CIP action on the survival curve.

## 5. Conclusions

Exposure to CO_2_, the toxicity of which has been investigated for almost a century, can alter the acid/base balance and cellular metabolism. Studying the effects of long-term exposure to CO_2_ is important because of the proven ability of this compound, in addition to H_2_O_2_, to cause mutagenesis in bacteria [28]. The presence of CO_2_ significantly delayed the bactericidal effect of CIP against *E. coli*. The assays showed that the formation of ROS was reduced when CIP was applied under CO_2_ conditions compared to AC. In contrast, RNS levels increased significantly under lower CO_2_ concentrations in the presence of CIP, while a similar synergistic effect was not observed at higher CO_2_ concentrations. The interaction between CIP and CO_2_ affected enzymatic and non-enzymatic antioxidant activities differently at varying CO_2_ concentrations. Specifically, at AC, CIP increased both SOD and CAT activities, but this response was diminished at higher CO_2_ levels. The GSH antioxidant capacity displayed variable responses based on CIP concentration and CO_2_ levels. The presence of CO_2_ alone caused a marked decrease in the antioxidant capacity in *E. coli*. These conclusions highlight the complex interplay between antibiotic efficacy, oxidative stress responses, and environmental conditions such as CO_2_ concentration in bacterial survival and behavior. According to the results obtained in this work, it is important to evaluate the effects of exogenous CO_2_ on the oxidative stress generated by antibiotics such as CIP. In summary, our findings indicate that the effect of CO_2_ on oxidative stress mediated by CIP in bacterial cells has implications not only for the environment, but also for human health.

## Figures and Tables

**Table 1:** Nitric oxide determination via Griess reaction in *E. coli* ATCC 25922.

	AC	CO_2_ 50 ppm	CO_2_ 50,000 ppm
Control	75.577 ± 0.024	74.748 ± 0.040	86.377 ± 0.015
CIP 0.5 µg/mL	73.238 ± 0.003	84.985 ± 0.011 *^#^	83.174 ± 0.010 ^#^
CIP 50 µg/mL	73.788 ± 0.003	81.921 ± 0.003 *^#^	84.567 ± 0.012 ^#^

Values are expressed in µM of NaNO_2_ per mg protein. * *p* < 0.05 relative to control in each condition. # *p* < 0.05 between CIP and CO_2_ concentrations.

**Table 2:** Ferric reducing antioxidant power (FRAP) determination in *E. coli* ATCC 25922.

	ACs	CO_2_ 50 ppm	CO_2_ 50,000 ppm
Control	126.317 ± 0.003	35.936 ± 0.003 *	86.678 ± 0.006 *
CIP 0.5 µg/mL	44.711 ± 0.001	42.303 ± 0.003	39.700 ± 0.016
CIP 50 µg/mL	43.011 ± 0.007	43.346 ± 0.001	42.508 ± 0.001

Values are expressed in µM of Fe^2+^ per mg protein. * *p* < 0.05 compared to the control in each condition.

## Data Availability

The data presented in this study are available on request from the corresponding author due to privacy.

## Supplementary Materials

The following supporting information can be downloaded at https://www.mdpi.com/article/10.3390/pathogens14070689/s1; Figure S1: Scheme of the experimental equipment setup for the incubation of *E. coli*; Figure S2: Effect of Tyron as ROS scavenger (A); Figure S3: Effect of Tyron as ROS scavenger (B); Figure S4: Effect of 2,2′-bipyridyl as ROS scavenger (A); Figure S5: Effect of 2,2′-bipyridyl as ROS scavenger (B); Figure S6: Effect of CPTIO as RNI scavenger; Figure S7: Effect of Tyron as RNI scavenger. References [47,48,49,50,51,52,53,54,55,56] are cited in Supplementary Materials.

## Abbreviations

The following abbreviations are used in this manuscript:

ACs	Atmospheric conditions
a.u	Arbitrary fluorescence units
CAT	Catalase
CFU	Colony-forming units
CIP	Ciprofloxacin
CO_2_	Carbon dioxide
DNA	Deoxyribonucleic acid
FRAP	Ferric reducing assay potency
GSH	Reduced glutathione
HO^•^	Hydroxyl radical
H_2_O_2_	Hydrogen peroxide
H_2_-DCFDA	2′,7′-Dichlorodihydrofluorescein diacetate
LB	Luria–Bertani
NBT	Nitro blue tetrazolium
HCO_3_^−^	Bicarbonate
NO	Nitric oxide
OD	Optical density
PBS	Phosphate-buffered saline
ROS	Reactive oxygen species
O_2_^•−^	Superoxide anion
RNS	Reactive nitrogen species
SD	Standard deviation
SOD	Superoxide dismutase
U	Unit

## References

[1] World Meteorological Organization (WMO) GREENHOUSE GAS BULLETIN. The State Greenhouse in the Atmosphere Based on Global Observations Through 2016. 12 November 2018. https://library.wmo.int/doc_num.php?explnum_id=4022.

[2] Butler J.H., Montzka S.A. The NOAA Annual Greenhouse Gas Index (AGGI). 12 November 2018. https://www.esrl.noaa.gov/gmd/aggi/aggi.html.

[3] Walker H.H. (1932). Carbon dioxide as a factor affecting lag in bacterial growth. Science.

[4] Ezraty B., Chabalier M., Ducret A., Maisonneuve E., Dukan S. (2011). CO_2_ exacerbates oxygen toxicity. EMBO Rep..

[5] Martínez H., Buhse T., Rivera M., Parmananda P., Ayala G., Sánchez J. (2012). Endogenous CO_2_ may inhibit bacterial growth and induce virulence gene expression in enteropathogenic *Escherichia coli*. Microb. Pathog..

[6] Cummins E.P., Selfridge A.C., Sporn P.H., Sznajder J.I., Taylor C.T. (2014). Carbon dioxide-sensing in organisms and its implications for human disease. Cell. Mol. Life Sci..

[7] Coakley R.J., Taggart C., Greene C., McElvaney N.G., O’Neill S.J. (2002). Ambient pCO_2_ modulates intracellular pH, intracellular oxidant generation, and interleukin-8 secretion in human neutrophils. J. Leukoc. Biol..

[8] Abolhassani M., Guais A., Chaumet-Riffaud P., Sasco A.J., Schwartz L. (2009). Carbon dioxide inhalation causes pulmonary inflammation. Am. J. Physiol. Lung Cell. Mol. Physiol..

[9] Karsten V., Murray S.R., Pike J., Troy K., Ittensohn M., Kondradzhyan M., Low K.B., Bermudes D. (2009). *msbB* deletion confers acute sensitivity to CO_2_ in *Salmonella enterica* serovar Typhimurium that can be suppressed by a loss-of-function mutation in zwf. BMC Microbiol..

[10] Visca P., Fabozzi G., Milani M., Bolognesi M., Ascenzi P. (2002). Nitric oxide and *Mycobacterium leprae* pathogenicity. IUBMB Life.

[11] Storz G., Imlay J.A. (1999). Oxidative stress. Curr. Opin. Microbiol..

[12] Imlay J.A. (2008). Cellular defenses against superoxide and hydrogen peroxide. Annu. Rev. Biochem..

[13] Hochgrafe F., Wolf C., Fuchs S., Liebeke M., Lalk M., Engelmann S., Hecker M. (2008). Nitric oxide stress induces different responses but mediates comparable protein thiol protection in *Bacillus subtilis* and *Staphylococcus aureus*. J. Bacteriol..

[14] Overton T.W., Justino M.C., Li Y., Baptista J.M., Melo A.M.P., Cole J.A., Saraiva L.M. (2008). Widespread distribution in pathogenic bacteria of Di-Iron proteins that repair oxidative and nitrosative damage to Iron-Sulfur centers. J. Bacteriol..

[15] Storz G., Zheng M., Storz G., Hengge-Aronis R. (2000). Oxidative stress. Bacterial Stress Responses.

[16] Albesa I., Becerra M.C., Battán P.C., Páez P.L. (2004). Oxidative stress involved in the antibacterial action of different antibiotics. Biochem. Bioph Res. Commun..

[17] Mustaev A., Malik M., Zhao X., Kurepina N., Luan G., Oppegard L.M., Hiasa H., Marks K.R., Kerns R.J., Berger J.M. (2014). Fluoroquinolone-gyrase-DNA complexes TWO MODES OF DRUG BINDING. J. Biol. Chem..

[18] Becerra M.C., Sarmiento M., Páez P.L., Arguello G., Albesa I. (2004). Light effect and reactive oxygen species in the action of ciprofloxacin on *Staphylococcus aureus*. J. Photochem. Photobiol. B.

[19] Becerra M.C., Páez P.L., Larovere L.E., Albesa I. (2006). Lipids and DNA oxidation in *Staphylococcus aureus* as a consequence of oxidative stress generated by ciprofloxacin. Mol. Cell Biochem..

[20] Quinteros M.A., Aiassa Martínez I.M., Paraje M.G., Dalmasso P.R., Páez P.L. (2016). Silver nanoparticles: Biosynthesis using an ATCC reference strain of *Pseudomonas aeruginosa* and activity as broad spectrum clinical antibacterial agents. Int. J. Biomater..

[21] Martínez S.R., Miana G.E., Albesa I., Mazzieri M.R., Becerra M.C. (2016). Evaluation of antibacterial activity and reactive species generation of n-benzenesulfonyl derivatives of heterocycles. Chem. Pharm. Bull..

[22] Chen X., Zhong Z., Xu Z., Chen L., Wang Y. (2010). 2′,7′-dichlorodihydrofluorescein as a fluorescent probe for reactive oxygen species measurement: Forty years of application and controversy. Free Radic. Res..

[23] Quinteros M.A., Cano Aristizábal V., Dalmasso P.R., Paraje M.G., Páez P.L. (2016). Oxidative stress generation of silver nanoparticles in three bacterial genera and its relationship with the antimicrobial activity. Toxicol. Vitr..

[24] Martínez S.R., Aiassa V., Becerra M.C. (2020). Oxidative stress response in reference and clinical Staphylococcus aureus strains under Linezolid exposure. J. Glob. Antimicrob. Re.

[25] Peralta M.A., da Silva M.A., Ortega M.G., Cabrera J.L., Paraje M.G. (2017). Usnic acid activity on oxidative and nitrosative stress of azole-resistant *Candida albicans* biofilm. Planta Med..

[26] Páez P.L., Becerra M.C., Albesa I. (2009). Antioxidative mechanisms protect resistant strains of *Staphylococcus aureus* against ciprofloxacin oxidative damage. Fundam. Clin. Pharmacol..

[27] Aiassa V., Barnes A.I., Albesa I. (2010). Resistance to ciprofloxacin by enhancement of antioxidant defenses in biofilm and planktonic *Proteus mirabilis*. Biochem. Biophys. Res. Commun..

[28] Páez P.L., Becerra M.C., Albesa I. (2008). Chloramphenicol induced oxidative stress in Human neutrophils. Basic Clin. Pharmacol. Toxicol..

[29] Páez P.L., Becerra M.C., Albesa I. (2010). Effect of the association of reduced glutathione and ciprofloxacin on the antimicrobial activity in *Staphylococcus aureus*. FEMS Microbiol. Lett..

[30] Farha M.A., French S., Stokes J.M., Brown E.D. (2018). Bicarbonate alters bacterial susceptibility to antibiotics by targeting the proton motive force. ACS Infect. Dis..

[31] Bauernfeind A., Petermtiller C. (1983). In Vitro activity of ciprofloxaein, norfloxacin and nalidixic acid. Eur. J. Clin. Microbiol..

[32] Zeiler H.J., Grohe K., Neu H.C., Reeves D.S. (1986). The In Vitro and In Vivo activity of ciprofloxacin. Ciprofloxacin.

[33] Aagaard J., Gasser T., Rhodes E., Madsen E.O. (1991). MICs of Ciprofloxacin and Trimethoprim for Escherichia coli: Influence of pH, lnoculum Size and Various Body Fluids. Infection.

[34] Borrell J.H., Montero M.T. (1997). Calculating Microspecies Concentration of Zwitterion Amphoteric Compounds: Ciprofloxacin as Example. J. Chem. Educ..

[35] De Bel E., Dewulf J., De Witte B., Van Langenhove H., Janssen C. (2009). Influence of pH on the sonolysis of ciprofloxacin: Biodegradability, ecotoxicity and antibiotic activity of its degradation products. Chemosphere.

[36] Mitscher L.A., Ma Z., Ronald A.R., Low D.E. (2003). Structure-activity relationships of quinolones. Fluoroquinolone Antibiotics. Milestones in Drug Therapy.

[37] Páez P.L., Becerra M.C., Albesa I. (2011). Comparison of macromolecular oxidation by reactive oxygen species in three bacterial genera exposed to different antibiotics. Cell Biochem. Biophys..

[38] Quinteros M.A., Cano Aristizabal V., Onnainty R., Mary V.S., Theumer M.G., Granero G.E., Paraje M.G., Páez P.L. (2018). Biosynthesized silver nanoparticles: Decoding their mechanism of action in *Staphylococcus aureus* and *Escherichia coli*. Int. J. Biochem. Cell Biol..

[39] Aiassa V., Barnes A.I., Albesa I. (2014). Macromolecular oxidation in planktonic population and biofilms of *Proteus mirabilis* exposed to ciprofloxacin. Cell Biochem. Biophys..

[40] Masadeh M., Alzoubi K., Al-azzam S., Khabour O., Al-buhairan A. (2016). Ciprofloxacin induced antibacterial activity is atteneuated by pretreatment with antioxidant agents. Pathogens.

[41] Wink D.A., Mitchell J.B. (1998). Chemical biology of nitric oxide: Insights into regulatory, cytotoxic, and cytoprotective mechanisms of nitric oxide. Free Radic. Biol. Med..

[42] King P.A., Anderson V.E., Edwards J.O., Gustafson G., Plumb R.C., Suggs J.W. (1992). A stable solid that generates hydroxyl radical upon dissolution in aqueous solutions: Reaction with proteins and nucleic acid. J. Am. Chem. Soc..

[43] Salgo M.G., Stone K., Squadrito G.L., Battista J.R., Pryor W.A. (1995). Peroxynitrite causes DNA nicks in plasmid pBR322. Biochem. Biophys. Res. Commun..

[44] Goswami M., Mangoli S.H., Jawali N. (2006). Involvement of reactive oxygen species in the action of ciprofloxacin against *Escherichia coli*. Antimicrob. Agents Chemother..

[45] Becerra M.C., Albesa I. (2002). Oxidative stress induced by ciprofloxacin in *Staphylococcus aureus*. Biochem. Biophys. Res. Commun..

[46] Goswami M., Subramanian M., Kumar R., Jass J., Jawali N. (2016). Involvement of antibiotic efflux machinery in Glutathione-mediated decreased ciprofloxacin activity in *Escherichia coli*. Antimicrob. Agents Chemother..

[47] Dyachenko V., Rueckschloss U., Isenberg G. (2009). Modulation of cardiac mechanosensitive ion channels involves superoxide, nitric oxide and peroxynitrite. Cell Calcium.

[48] Marcén M., Ruiz V., Serrano M.J., Condón S., Mañas P. (2017). Oxidative stress in *E. coli* cells upon exposure to heat treatments. Int. J. Food Microbiol..

[49] De Alencar T.A.M., Wilmart-Gonçalves T.C., Vidal L.S., Fortunato R.S., Leitão A.C., Lage C. (2014). Bipyridine (2,2′-dipyridyl) potentiates *Escherichia coli* lethality induced by nitrogen mustard mechlorethamine. Mutat. Res. Fundam. Mol. Mech. Mutagen..

[50] Yu Q., Zhang B., Li J., Zhang B., Wang H., Li M. (2016). Endoplasmic reticulum-derived reactive oxygen species (ROS) is involved in toxicity of cell wall stress to *Candida albicans*. Free Radic. Biol. Med..

[51] Keshavarz-Tohid V., Taheri P., Taghavi S.M., Tarighi S. (2016). The role of nitric oxide in basal and induced resistance in relation with hydrogen peroxide and antioxidant enzymes. J. Plant Physiol..

[52] Galera I.L.D., Paraje M.G., Páez P.L. (2016). Relationship between oxidative and nitrosative stress induced by gentamicin and ciprofloxacin in bacteria. J. Biol. Nat..

[53] Augusto O., Bonini M.G., Amanso A.M., Linares E., Santos C.C.X., De Menezes S.L. (2002). Nitrogen dioxide and carbonate radical anion: Two emerging radicals in biology. Free Radic. Biol. Med..

[54] Goldstein S., Czapski G. (1998). Formation of peroxynitrate from the reaction of peroxynitrite with CO_2_: Evidence for carbonate radical production. J. Am. Chem. Soc..

[55] Bonini M.G., Radi R., Ferrer-Sueta G., Ferreira A.M.D.C., Augusto O. (1999). Direct EPR detection of the carbonate radical anion produced from peroxynitrite and carbon dioxide. J. Biol. Chem..

[56] Kuwahara H., Miyamoto Y., Akaike T., Kubota T., Sawa T., Okamoto S., Maeda H. (2000). *Helicobacter pylori* urease suppresses bactericidal activity of peroxynitrite via carbon dioxide production. Infect. Immun..

