# Supplementary material for: Atmospheric Carbon Dioxide Modifies the Antimicrobial Activity and Oxidative Stress Generated by Ciprofloxacin in Escherichia coli
Source: Pathogens. 2025 Jul 14;14(7):689. doi: 10.3390/pathogens14070689 (PMC12300494; doi:10.3390/pathogens14070689)
Supplement: Supplementary file 1 [file pathogens-14-00689-s001.zip › pathogens-3726589-supplementary.pdf]

SUPPLEMENTARY MATERIAL

Atmospheric carbon dioxide modifies the antimicrobial activity and oxidative stress generated by ciprofloxacin in *Escherichia coli*

Cano Aristizábal V[1,3], Mendoza Ocampo ES[1,3], Quinteros MA[1,4], Paraje MG[2,4] and Páez PL[1,3*]

[1]Dto. Ciencias Farmacéuticas, Facultad de Ciencias Químicas, Universidad Nacional de Córdoba, Argentina.

[2]Cátedra de Microbiología, Facultad de Ciencias Exactas Físicas y Naturales, Universidad Nacional de Córdoba, Argentina.

[3]Unidad de Investigación y Desarrollo en Tecnología Farmacéutica (UNITEFA), Consejo Nacional de Investigaciones Científicas y Técnicas (CONICET), Argentina

[4]Instituto Multidisciplinario de Biología Vegetal (IMBIV), Consejo Nacional de Investigaciones Científicas y Técnicas (CONICET), Argentina.

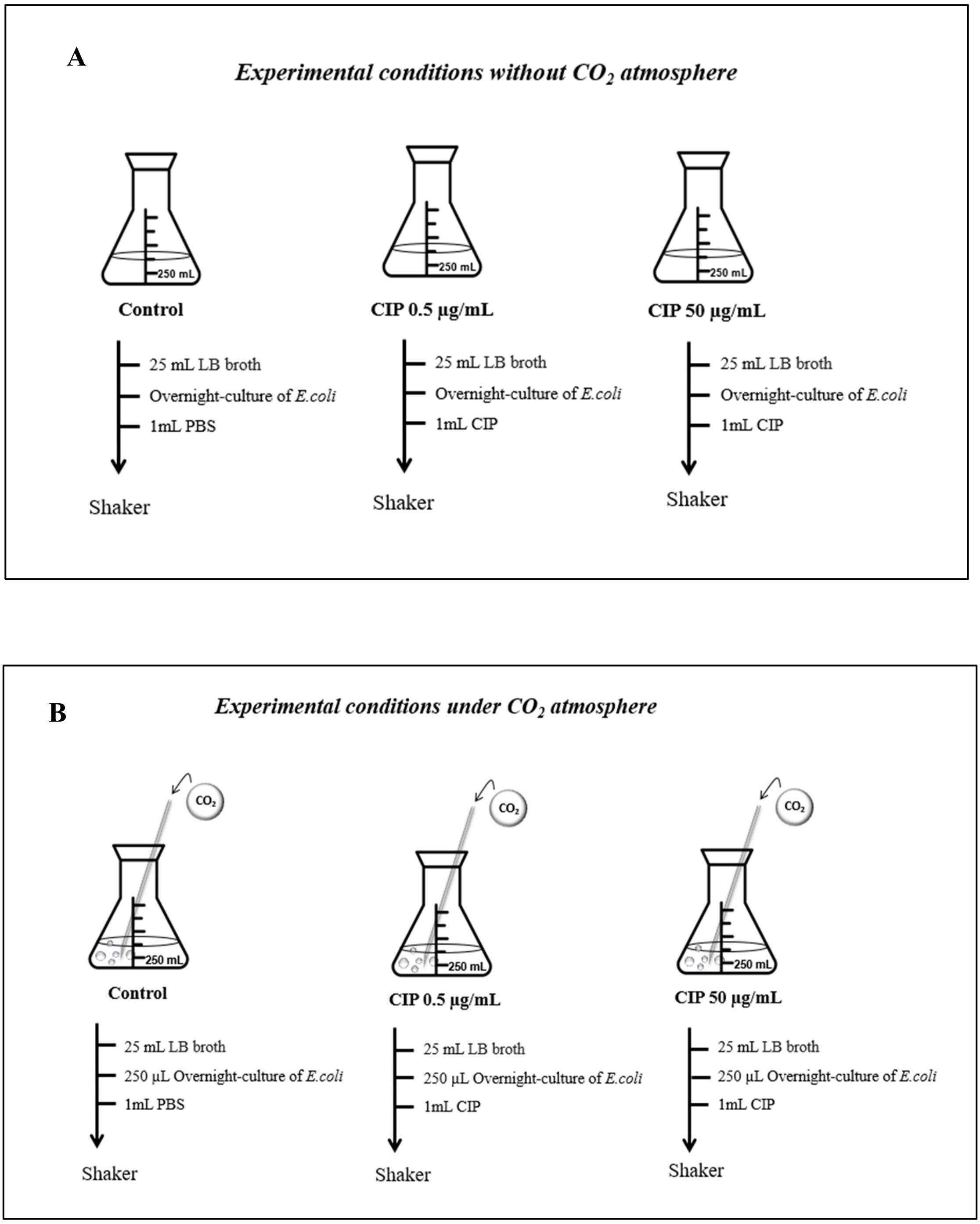

Figure S1. Scheme of the experimental equipment setup for the incubation of *E. coli* A) without and B) with CO_2 addition.

The experimental design follows the same line describe in ROS and RNI determinations shown in the Materials and Methods section of the MS. The only difference was the addition of 1 mL of the selected scavengers before the incubation time. The used scavengers were Tyron 10mM (Sigma), 2,2'-bipyridyl 250 µM (Sigma), and carboxy-PTIO 100 µM (CPTIO, Sigma). The obtained values are shown as a.u ROS/CFU.mL^{-1} or a.u of ROS/mg of protein. The mg of ROS formation was related to the respective control condition.

The obtained results confirm that, when the ROS and RNI formation pathways are blocked by Tyron[(47,48)], 2,2'-bipyridyl[(49,50)], and CPTIO[(51)], the CO_2 induced the apparition of a new pathway for the formation of these species, independently of CIP and CO_2 concentrations.

At ACs and in the presence of CIP, $O_2^{\bullet-}$ was the most expressed species at 2h of incubation time. This result may be due to $O_2^{\bullet-}$ because it is the first compound to be formed (see Figures S1 and S2). The obtained results, in the presence of Tyron and CPTIO, were proof that NO and $O_2^{\bullet-}$ participation was low (Figures S6 and S7), indicating that CIP, at ACs, does not favor RNI formation. The results mentioned before are in agreement with other results reported in the literature, where it has been shown that CIP generates oxidative stress in *E. coli* and *S. aureus* through an increase in $O_2^{\bullet-}$ at a short time and independently of CIP concentration. For this reason, the ROS increment reduces the NO levels due to the cross-reaction between them[(44,45,37,52)]. At the same time, Masadeh et al. showed that the ROS formed, induced by CIP in *E. coli*, are $O_2^{\bullet-}$ and singlet oxygen ($^1O_2$). Also, they reported that this effect was inhibited by previous treatment, in bacteria, with antioxidant agents such as tempol, melatonin, pentoxifylline, and vitamins C and E[(40)].

On the other hand, in the presence of CO_2 and the scavengers mentioned before, a change was observed, compared to ACs, in the formed species induced by CIP (Figures S1, 2, 3, and 4), with $HO^\bullet$ and NO being the species that were most increased in those conditions.

The changes observed in ROS and RNI formation may be due to the perturbed equilibrium between the internal and external CO_2 concentrations, which favored HCO_3^- and CO_3^{-2} formation. Those results are in agreement with the study of Ezraty et al., where they showed that both HCO_3^- and CO_3^{-2} suffer fast oxidations by $HO^{\bullet}$ to form $CO_3^{\bullet -}$, with the rate coefficients for the reactions being k = 8.5x10^6 and 3.0x10^8 $M^{-1}s^{-1}$, respectively. Also, that study takes into account the fact that the formation rate of $HO^{\bullet}$ (k = 3.0x10^8 $M^{-1}s^{-1}$) is much lower than the formation rate of $CO_3^{\bullet -}$; thus, the researchers believe that the decrease in ROS formation could be due to a fast consumption of $HO^{\bullet}$, which fails to be compensated by the formation rate[(50,53)].

Also, the high NO participation observed in this work at CO_2 conditions favored the production of peroxynitrite ion ($ONOO^-$); moreover, it has been described that this anion has the ability to react with CO_2 to form the nitroperoxycarbonate anion ($ONOOCO_2^-$) with a rate coefficient of k = 3.1x10^4 $M^{-1}s^{-1}$ [(54)], where hydrolysis is the most probable reactive pathway with a yield of 60%, producing CO_3^{-2} and NO_2[(55)]. Thus, the CO_2 effect will consist in preventing nitration and oxidative damage. This effect was observed by Kuwahara et al.[(56)], who proved that CO_2 inhibits the cytotoxic effect of $ONOO^-$ in *Helicobacter pylori.*

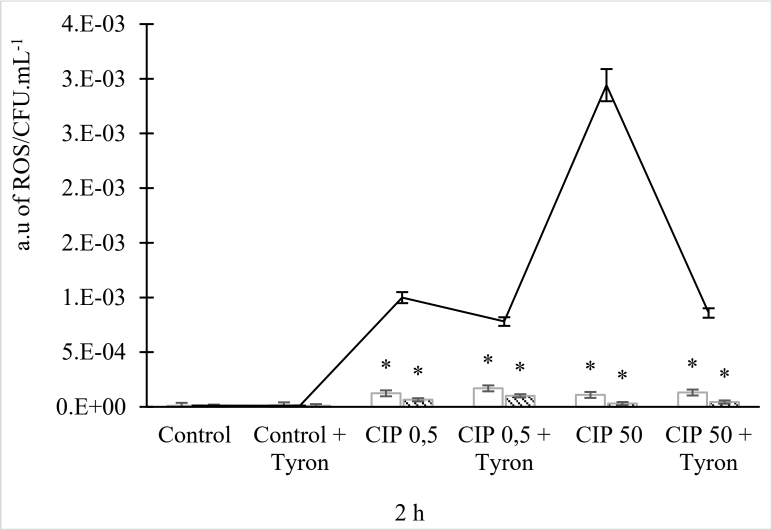

Figure S2. Effect of Tyron as ROS scavenger (A). (—) ACs; (□) CO_2 50 ppm; ( ) CO_2 50,000 ppm. The assays were carried out in triplicate. The line and bars represent ROS formation in percent at each condition. The error bars represent *$p<0.05$ with respect to ACs.

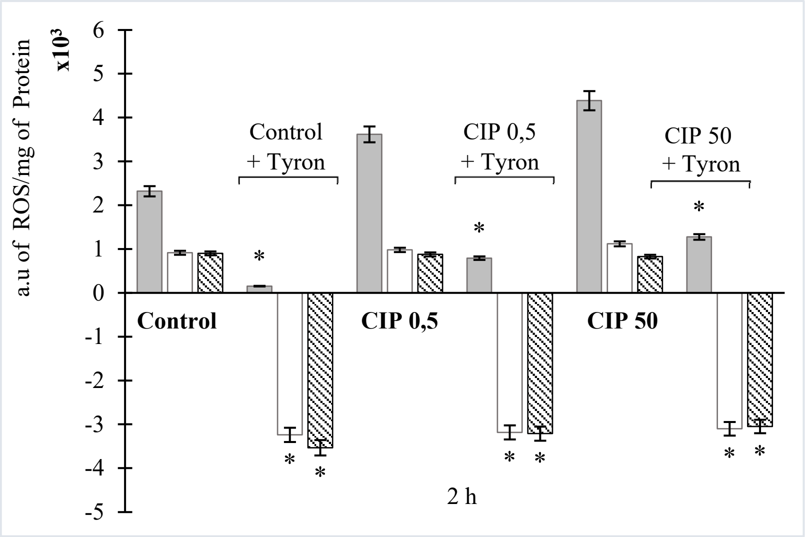

Figure S3. Effect of Tyron as ROS scavenger (B). (▪) ACs; (□) CO_2 50 ppm; ( ) CO_2 50,000 ppm. The assays were carried out in triplicate. The bars represent ROS formation in percent at each condition, in the presence or absence of Tyron. The error bars represent *$p<0.05$ with respect to the absence of Tyron.

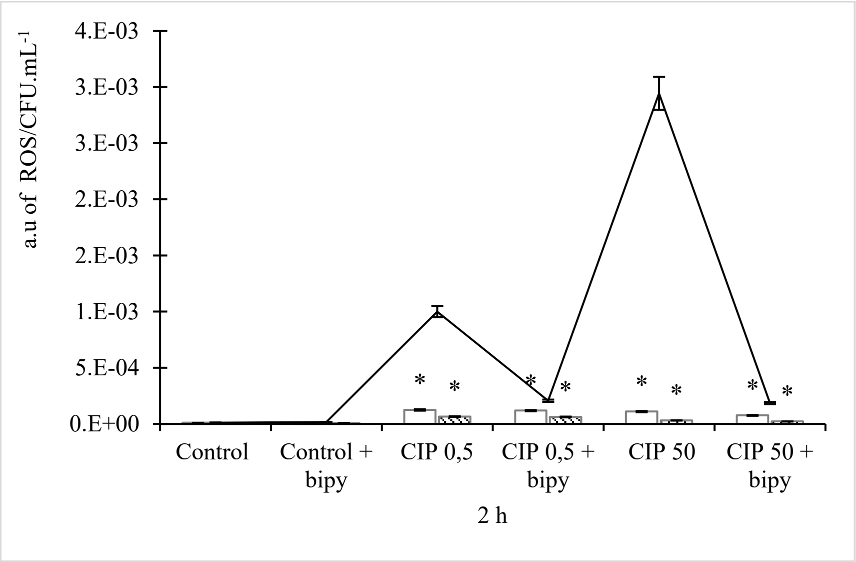

Figure S4. Effect of 2,2'-bipyridyl as ROS scavenger (A). (—) ACs; (□) CO_2 50 ppm; ( ) CO_2 50,000 ppm. The assays were carried out in triplicate. The line and bars represent ROS formation in percent at each condition. The error bars represent *$p<0.05$ with respect to ACs.

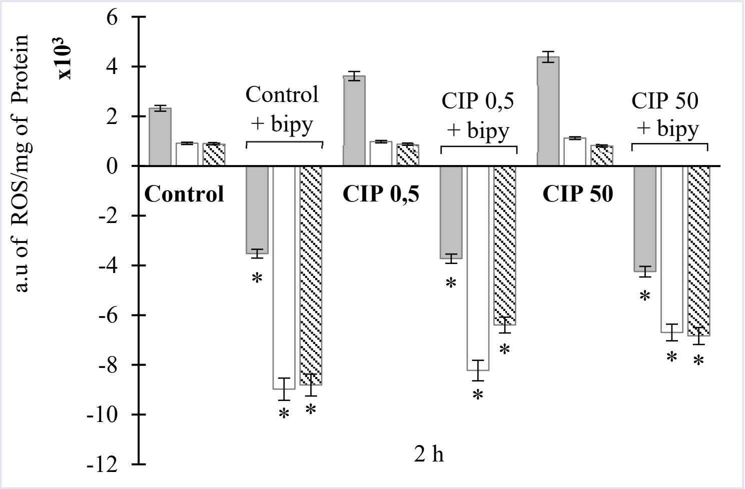

Figure S5. Effect of 2,2'-bipyridyl as ROS scavenger (B). (□) ACs; (□) CO_2 50 ppm; ( ) CO_2 50,000 ppm. The assays were carried out in triplicate. The bars represent ROS formation in percent at each condition, in the presence or absence of 2,2'-bipyridyl. The error bars represent *$p<0.05$ with respect to the absence of 2,2'-bipyridyl.

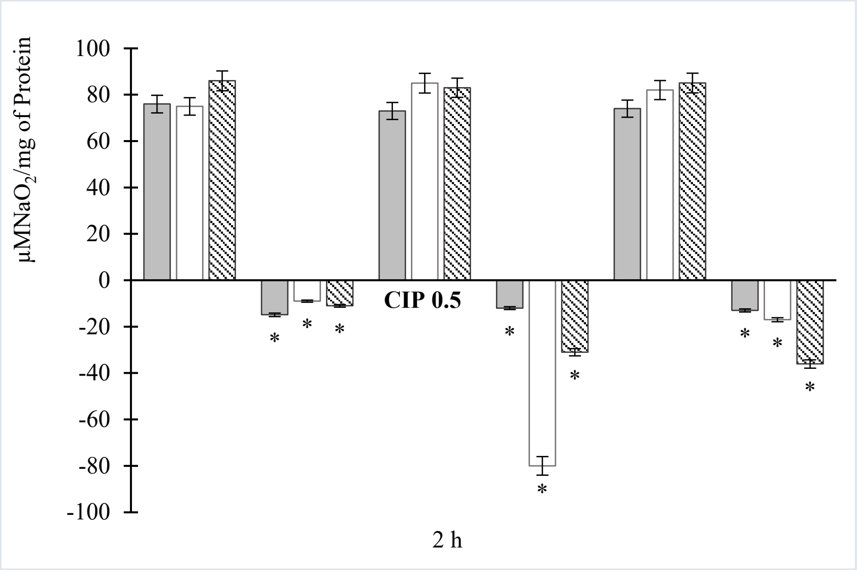

Figure S6. Effect of CPTIO as RNI scavenger. (▤) ACs; (□) CO_2 50 ppm; (▧) CO_2 50,000 ppm. The assays were carried out in triplicate. The bars represent RNI formation in percent at each condition, in the presence or absence of CPTIO. The error bars represent *p<0.05 with respect to the absence of CPTIO.

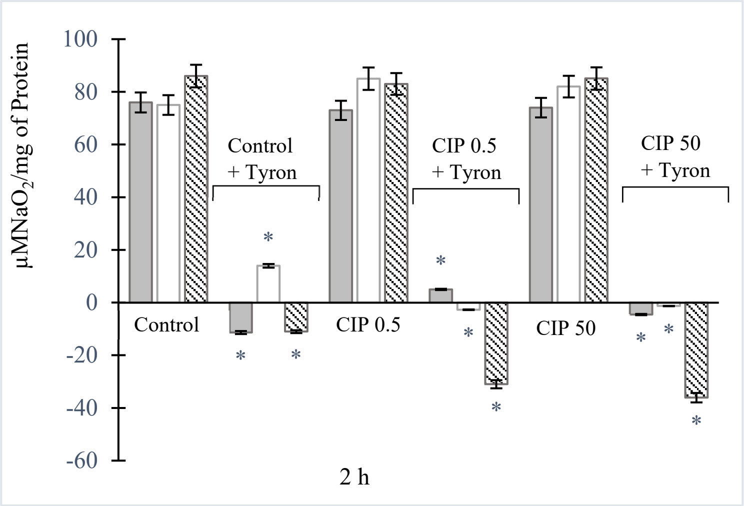

Figure S7. Effect of Tyron as RNI scavenger. (▤) ACs; (□) CO_2 50 ppm; (▧) CO_2 50,000 ppm. The assays were carried out in triplicate. The bars represent RNI formation in percent at each condition, in the presence or absence of Tyron. The error bars represent *p<0.05 with respect to the absence of Tyron.
